# *WDFY3* mutation alters laminar position and morphology of cortical neurons

**DOI:** 10.1186/s13229-022-00508-3

**Published:** 2022-06-22

**Authors:** Zachary A. Schaaf, Lyvin Tat, Noemi Cannizzaro, Ralph Green, Thomas Rülicke, Simon Hippenmeyer, Konstantinos S. Zarbalis

**Affiliations:** 1 University of California at Davis, Department of Pathology and Laboratory Medicine, Sacramento, CA 95817 USA; 2 Shriners Hospitals for Children Northern California, Sacramento, CA 95817 USA; 3 Department of Biomedical Sciences, University of Veterinary Medicine Vienna, 1210 Vienna, Austria; 4 Institute of Science and Technology Austria, Am Campus 1, 3400 Klosterneuburg, Austria; 5 UC Davis MIND Institute, Sacramento, CA 95817 USA

**Keywords:** WDFY3, Cerebral cortex, Neuronal migration, Excitatory neurons, Dendrites, Dendritic spines

## Abstract

**Background:**

Proper cerebral cortical development depends on the tightly orchestrated migration of newly born neurons from the inner ventricular and subventricular zones to the outer cortical plate. Any disturbance in this process during prenatal stages may lead to neuronal migration disorders (NMDs), which can vary in extent from focal to global. Furthermore, NMDs show a substantial comorbidity with other neurodevelopmental disorders, notably autism spectrum disorders (ASDs). Our previous work demonstrated focal neuronal migration defects in mice carrying loss-of-function alleles of the recognized autism risk gene *WDFY3*. However, the cellular origins of these defects in *Wdfy3* mutant mice remain elusive and uncovering it will provide critical insight into *WDFY3*-dependent disease pathology.

**Methods:**

Here, in an effort to untangle the origins of NMDs in *Wdfy3*^*lacZ*^ mice, we employed mosaic analysis with double markers (MADM). MADM technology enabled us to genetically distinctly track and phenotypically analyze mutant and wild-type cells concomitantly in vivo using immunofluorescent techniques.

**Results:**

We revealed a cell autonomous requirement of WDFY3 for accurate laminar positioning of cortical projection neurons and elimination of mispositioned cells during early postnatal life. In addition, we identified significant deviations in dendritic arborization, as well as synaptic density and morphology between wild type, heterozygous, and homozygous *Wdfy3* mutant neurons in *Wdfy3*-MADM reporter mice at postnatal stages.

**Limitations:**

While *Wdfy3* mutant mice have provided valuable insight into prenatal aspects of ASD pathology that remain inaccessible to investigation in humans, like most animal models, they do not a perfectly replicate all aspects of human ASD biology. The lack of human data makes it indeterminate whether morphological deviations described here apply to ASD patients or some of the other neurodevelopmental conditions associated with *WDFY3* mutation.

**Conclusions:**

Our genetic approach revealed several cell autonomous requirements of WDFY3 in neuronal development that could underlie the pathogenic mechanisms of *WDFY3*-related neurodevelopmental conditions. The results are also consistent with findings in other ASD animal models and patients and suggest an important role for WDFY3 in regulating neuronal function and interconnectivity in postnatal life.

**Supplementary Information:**

The online version contains supplementary material available at 10.1186/s13229-022-00508-3.

## Background

During prenatal neurogenesis, newly born neurons are deployed from proliferative compartments surrounding the ventricles towards the surface of the brain where they will settle into their proper laminae and nuclei to form functional circuits [1–4]. Any disturbance of this tightly orchestrated process can result in neuronal migration disorders (NMDs), birth defects with often devastating consequences for affected individuals [5–9]. In the cerebral cortex, the primary site of many NMDs, projection neurons are generated in the proliferative layers of the ventricular and subventricular zones (VZ, SVZ) from radial glial cells (RGCs) and migrate radially to the pial surface along radial glial fibers [2, 10, 11]. The earliest born neurons building the cortical plate become the deepest layer of the cortex, and ensuing waves of newly born neurons migrate past their predecessors forming layer upon layer below the superficial marginal zone. NMDs can range from relatively mild cases such as focal cortical dysplasia with only limited migration anomalies [12–14] to crippling diseases like lissencephaly, which display a reduction in gyri and a thickening of gray matter over a large area or the entire cerebral cortex often leading to death in early childhood [15, 16].

Interestingly, there is a pronounced comorbidity of NMDs with autism spectrum disorders (ASDs), a fact that may contribute to the high prevalence of epilepsy in ASDs with co-diagnoses reaching up to 40% of autism cases [17, 18]. Indeed, evidence from postmortem analysis of human cases [19–23] and more recently from ASD mouse models [24, 25] strongly suggests that focal cortical dysplasia is a common feature of the autistic brain, further strengthening the notion that a subset of autism cases is rooted in dysregulations of prenatal neurogenesis.

We previously reported on the generation of *Wdfy3* mutant mice and on the neurodevelopmental anomalies associated with Wdfy3 loss-of-function [24]. *WDFY3* is an autism risk gene and causative in a range of other often comorbid neurodevelopmental disorders, including neurodevelopmental delay, intellectual disability, and hypotonia [26–32]. Depending on the specific allele, heterozygous *WDFY3* loss in humans is typically associated with macrocephaly, a feature faithfully modeled in heterozygous and homozygous mutant mice. *Wdfy* genes (1–4) encode a small family of four neuronally expressed, intracellular molecules associated with vesicular transport. WDFY3 is a member of the BEACH (**be**ige **a**nd **CH**S proteins) protein family and contains, in addition to the BEACH domain, a PH domain, five WD40 domains, and a C-terminal FYVE (**F**ab1/**Y**OTB/**V**ac1/**E**EA1) domain enabling its integration into vesicular membranes [32, 33]. Human WDFY3 has been shown to act as an autophagosomal scaffolding protein required for the selective recruitment and degradation of macromolecular components such as aggregation-prone proteins. Untangling the cellular causes underlying the morphological abnormalities in *Wdfy3* mutant mice, we identified an essential role for WDFY3 in regulating a subset of RGC divisions by promoting asymmetric differentiative over symmetric proliferative divisions. Consequently, WDFY3 loss promotes self-renewing divisions in RGCs, transient expansion of the progenitor pool, increased neurogenesis, and larger brains. In addition, homozygous *Wdfy3* mutation in mice results in focal neuronal migration defects in which deep-layer neurons are typically mispositioned within upper layers.

Radial migration of newly born cortical neurons is regulated by factors both intrinsic to the migrating cells and/or extrinsic, determined by the environment they navigate. Cell autonomous effectors may involve cytoskeletal proteins that control cellular motility, such as neuronal tubulins [33] and their regulators [34, 35], while cell non-autonomous effects may involve chemoattractants expressed by surrounding cells, such as reelin [36]. Earlier findings of WDFY3’s cytosolic and possibly vesicular localization within dividing progenitors suggested cell autonomous control over neuronal migration, but in the absence of functional evidence remained speculative.

Here, we employed mosaic analysis with double markers (MADM) [37, 38] to trace the laminar positioning of cortical projection neurons depending on *Wdfy3* genotype and quantitatively assess mispositioned cells to endogenous *Wdfy3* loss. In addition, we addressed the question of whether *Wdfy3* mutation has effects on neuronal morphology and circuit integration by evaluating dendritic arborization, spine density, and morphology. Our results demonstrate a predominantly cell autonomous role of *Wdfy3* in regulating either aspect of neuronal laminar and circuit integration.

## Methods

### Animal husbandry and genotyping

Animals were housed in Plexiglas cages (55 × 33 × 19 cm) and maintained in facilities approved by the Association for Assessment and Accreditation of Laboratory Animal Care (AAALAC) International under standard laboratory conditions (21 ± 2 °C; 55 ± 5% humidity) on a 12-h light/dark cycle, with ad libitum access to both water and standard rodent chow. Animal handling protocols were approved by the University of California at Davis Institutional Animal Care and Use Committee overseen by the AAALAC International accreditation program (latest accreditation in February 14, 2020) and in compliance with the ARRIVE [39] and NIH guidelines [40].

Mice carrying the *Wdfy3*^*lacZ*^* (Wdfy3*^*tm1a(KOMP)Mbp*^*)* allele were generated and genotyped as previously described [24] and maintained on C57BL/6NJ background. To generate *Wdfy3*^*lacZ*^-MADM-5 mice, we crossed *Wdfy3*^+*/lacZ*^ with homozygous MADM-5^*GT/GT*^ mice [38, 41]. Subsequently, compound heterozygous offspring was crossed with homozygous MADM-5^*TG/TG*^; *Emx1*^*Cre*^ mice. The resulting offspring was genotyped for the presence of *Wdfy3*^*lacZ*^, *Emx1*^*Cre*^, *GT*, and *TG* cassettes and once pups positive for all markers identified they were processed for histological analysis.

### Perfusion, brain collection, tissue preservation, and sectioning

Mice collected for histological analysis were transcardially perfused with phosphate-buffered saline (PBS) followed by 4% paraformaldehyde (PFA) in PBS using a medical pump at a rate of 1 mL/min and immersed in 4% PFA/PBS overnight at 4 °C. The next day skulls were washed with PBS, brains excavated, cryopreserved through gradual sucrose immersion (15%, 30% in PBS), embedded in O.C.T. compound (Fisher Healthcare), flash frozen on dry ice, and then transferred to − 80 °C until sectioning. O.C.T.-embedded brains were coronally sectioned on a Leica CM-1950 cryostat at a thickness of 30 μm. Sections were transferred onto glass slides (Thermo Fisher Scientific, Waltham, MA) and placed on a slide warmer (Premiere XH-2004) at 40 °C for a minimum of 36 h. Slides from the warmer were then placed in a slide box and frozen at − 80 °C until used.

Alternatively, dissected brains used for analysis of cell morphology were transferred into PBS/0.1% NaN_3_ until embedded in 4% agarose/PBS for immediate vibratome sectioning. Agarose-embedded brains were submerged in ice-cold PBS and coronally sectioned at 200 μm on a Leica VT-1000S vibratome. Sections were then transferred into 24-well plates with 1 mL of PBS on ice and stored at 4 °C until use.

### Immunofluorescent labeling

Slide-mounted sections were fixed with 4% PFA/PBS, followed by three washes in PBS for 5 min each. For antigen retrieval, slides were submerged in 1 × working concentration of Diva Decloaker solution (Biocare Medical, Pacheco, CA) while heated in a Decloaking Pressure Chamber at 90 °C for 30 min. Following this treatment, slides were washed in PBS three times for 5 min and tissue blocked with 10% donkey serum in PBS with 1% Triton X-100 (PBST) for 1 h at room temperature. After being washed with PBS, sections were incubated with chosen primary antibodies for 18 h at 4 °C, subsequently washed five times for 10 min each with PBS, and secondary antibodies applied for 2 h at room temperature. A list of all antibodies used in this study is provided in Table 1. After secondary incubation, slides were washed five times for 10 min each in PBS and for 5 min submerged in a 0.1% 4′,6-diamidino-2-phenylindole (DAPI) (Thermo Fisher Scientific) to create a nuclear counterstain. After being washed twice 10 min each with PBS, sections were covered with Fluoromount-G (Thermo Fisher Scientific), mounted with cover glass (Thermo Fisher Scientific), and then allowed to dry in a dark ventilated area for a minimum of 20 h before being imaged.

**Table 1:** Primary and secondary antibodies used

Target	Host species	Manufacturer	Catalog #	Dilution
GFP	Chicken	Aves Labs	GFP-1010	1:200
RFP	Rabbit	Medical and Biological Laboratories	PM005	1:100
MCherry	Goat	Biorbyt	ORB11618	1:300
BRN2	Rabbit	GeneTex	GTX114650	1:200
CTIP2	Rat	Abcam	AB18465	1:200
TBR1	Rabbit	Abcam	AB31940	1:200
Cleaved CAS3	Rabbit	Cell Signaling	9661	1:100

Target species	Host species (conjugate)	Manufacturer	Catalog #	Dilution
Rat	Donkey (405)	Jackson ImmunoResearch	712-475-153	1:200
Chicken	Donkey (488)	Jackson ImmunoResearch	703-545-155	1:200
Goat	Donkey (594)	Invitrogen	A-11058	1:200
Rabbit	Donkey (594)	Invitrogen	A-21207	1:200
Rabbit	Donkey (647)	Invitrogen	A-31573	1:200
Rat	Donkey (647)	Abcam	AB150155	1:200

Agarose-embedded sections (200 μm) were immunostained free floating on an orbital shaker (Labnet) at 30 rpm using the process described above while omitting antigen retrieval. Transfer of sections into washes, blocking, and incubation liquids was done using a paintbrush. Antibody incubation times were extended to account for increased tissue thickness compared with cryosections with primary incubation applied for 64 h at 4 °C and secondary incubation for 18 h at 4 °C. Sections were finally washed 6 times for 45 min each.

### Tissue clearing

To increase clarity and microscope laser penetration, post immunostaining 200-μm-thick sections used for cell morphological analysis were immersed overnight at 4 °C in ultrafast optical clearing method (FOCM) solution; 30% (wt/vol) urea, 20% (wt/vol) D-sorbitol, and 5% (wt/vol) glycerol were dissolved in dimethyl sulfoxide (DMSO) [42]. The next day the sections were removed from the FOCM solution, transferred to microscope slides (Thermo Fisher Scientific), and surrounded with Fluoromount-G (Thermo Fisher Scientific) before coverslipping. Mounted slides were set to dry in the dark for a minimum of three days before imaging.

### Microscopy and image processing

To assess genotype distribution and laminar positioning, fluorescent confocal microscopy and image acquisition was performed on an inverted Nikon C1 microscope with associated software, typically at 10 × magnification. Images used for cell morphological analysis, including dendritic arbor and spine analysis, were obtained on a Nikon A1 microscope with associated software. In either case, we imaged sections under varying acquisition parameters for best possible comparisons of the two fluorophores (GFP and tdT). Subsequently, images were uploaded and cells were counted in NIS-Elements. Cellular morphology was analyzed in FIJI using the Simple Neurite Tracer plugin where further adjustments ensured even brightness and detail of GFP and tdT in grayscale.

### Statistical testing

All data were processed, analyzed, and graphical figures created in GraphPad Prism 9. To remove suspected outliers, data were initially processed with the ROUT outlier test (Q value = 1%). Applicable data were then tested for normality, variance, and standard deviation before analysis. Lamination analysis was done using Fisher’s exact test, population analysis was done using an unpaired Student’s *t* test, and Sholl profile analysis was statistically evaluated by two-way analysis of variance (ANOVA), followed by a Tukey’s multiple comparison test. Analysis of bouton density was done using a one-way ANOVA with multiple comparisons, while bouton subtype distribution analysis was done using a two-way ANOVA with multiple comparisons. When applicable and reasonable to add, data are reported as a mean with standard deviation. Bar graphs with replicates are reported as mean with standard error of the mean. Results were considered to be statistically significant if *p* ≤ 0.05. Individual data points largely correspond to replicates of one brain. The extent of significance between groups is indicated with one, two, three, or four asterisks if *p* values were equal to or less than 0.05, 0.01, 0.001, or 0.0001, respectively.

## Results

### *Wdfy3* mutant cells get disproportionally lost during early postnatal development in MADM brains

**Fig. 1:**
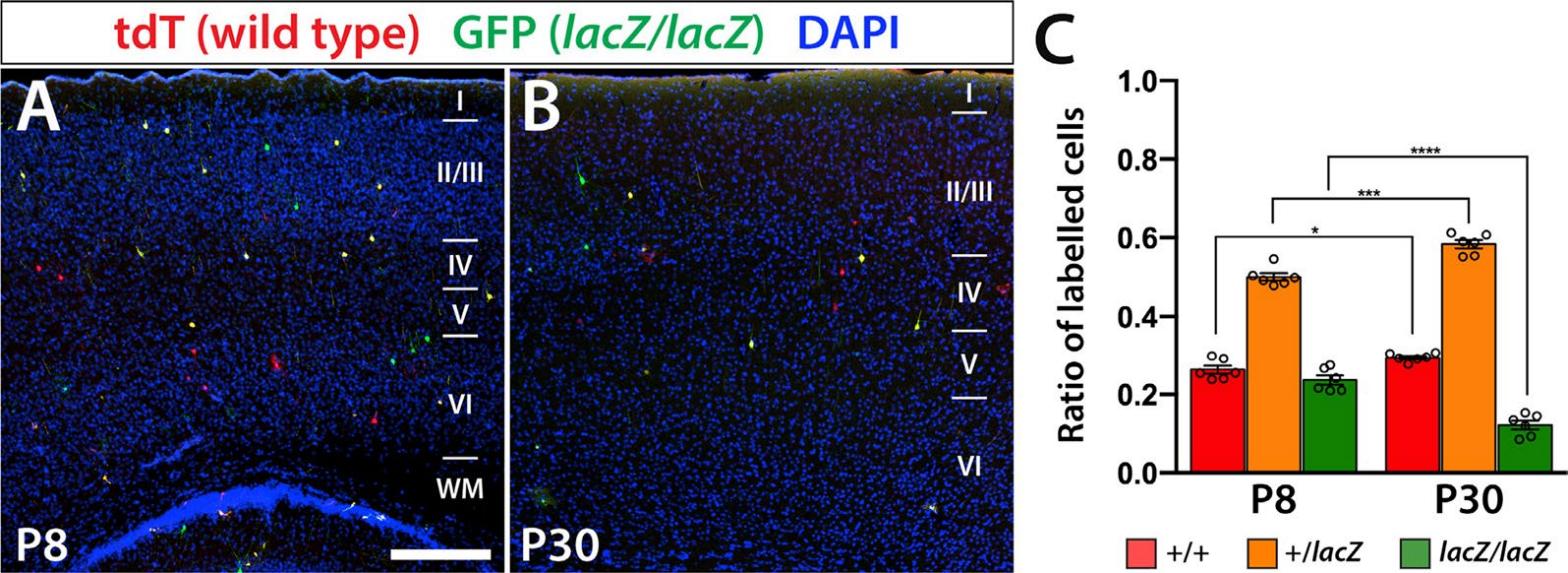
Genotype distributions in *Wdfy3*-MADM-5 cortex show specific loss of *Wdfy3*^*lacZ/lacZ*^ cells at early postnatal stages. **A**, **B** TdT and GFP immunofluorescence of somatosensory cortex at P8 (**A**) and P30 (**B**) show sparse neuronal and astrocytic labeling of expected genotypes, tdT^+^ cells are WT, GFP^+^ cells are *lacZ/lacZ*, and tdT^+^/GFP^+^ cells are heterozygous. The bar diagram in **C** depicts the changes in genotype distributions between the two developmental stages showing a significant relative reduction in GFP^+^ (*lacZ/lacZ*) neurons (*p* < 0.0001) and significant relative increases in tdT (WT; *p* = 0.02) and tdT^+^/GFP^+^ (+ /*lacZ*) neurons (*p* = 0.0002). Scale bar is 250 μm

To test the efficacy of the *Wdfy3*-MADM system, we obtained brains of mice carrying all four transgenic modifications (*Wdfy3*^*lacZ*^, MADM-5^*GT*^, MADM-5^*TG*^, and *Emx1*^*Cre*^) and immunolabeled for endogenous tdT and eGFP expression. We opted to analyze three male and three female brains each at two developmental stages, postnatal day (P)8, a time point at which developmental neurogenesis is completed and all cortical layers are formed and distinguished, and P30, a time point in which cell death associated with activity-dependent neuron elimination has largely faded [43]. After imaging, sections of both stages revealed the expected sparse labeling of neurons and astrocytes within all cortical layers with cells distributed across labels associated with the anticipated recombination of fluorophore encoding cassettes. We detected red tdT^+^ cells expected to be wild type (WT or + / +), green homozygous mutant GFP^+^ cells (*lacZ/lacZ*), and yellow heterozygous tdT^+^/GFP^+^ cells (+ */lacZ*) (Fig. 1A, B). We proceeded to count all cells in ten sections each of 3 male and 3 female brains per stage and converted the added numbers of each genotype to ratios of a whole. In total, we counted at P8 685 tdT^+^ cells, 1,330 tdT^+^/GFP^+^ cells, and 658 GFP^+^ cells and at P30 614 tdT^+^ cells, 1,236 tdT^+^/GFP^+^ cells, and 262 GFP^+^ cells. Genotype populations were compared across each other and between the two time points (P8 and P30). In P8 brains, the distribution of GFP labeled homozygous mutant neurons was ~ 24%, likely due to cell death significantly decreasing to ~ 12% in P30 brains (0.2368 ± 0.029 at P8 and 0.1216 ± 0.028 at P30; *p* < 0.0001) (Fig. 1C). This relative decrease in mutant cells at P30 was accompanied by a significant relative increase in both WT (0.2634 ± 0.026 at P8 and 0.2949 ± 0.010 at P30; *p* = 0.02) and + */lacZ* cells (0.4998 ± 0.024 at P8 and 0.5835 ± 0.026 at P30; *p* = 0.0002) (Fig. 1C). No significant differences between the sexes were observed.

To unveil direct evidence of increased apoptotic rates of *lacZ/lacZ* neurons, we performed cleaved CAS3 immunofluorescence at P8 with the aim to establish ratios of tdT^+^/CAS3^+^, GFP^+^/CAS3^+^, or tdT^+^/GFP^+^/CAS3^+^ cells and derive from these ratios statistically significant differences between genotypes. Labeling 40 sections of six brains with this approach and analyzing a total of ~ 1,800 neurons of all genotypes, we did not discover a single cell in which the GFP or tdT signal would overlap with the CAS3 signal. The sparsity of MADM-labeled cells in combination with the rarity of cell death in the postnatal brain presented a barrier to discovery, an inherent limitation of the approach used here (Additional file 1: Fig. S1).

### Cortical lamination errors occur more frequently in *Wdfy3* homozygous mutant cells

Realizing that early postnatal development is associated with the disproportionate loss of *lacZ/lacZ* neurons, we proceeded to examine whether this loss is associated with the laminar positioning of these neurons. We performed immunofluorescent labeling targeting endogenous tdT and eGFP expression produced as a result of the MADM system, and colabeled for cortical layer markers TBR1, CTIP2, and BRN2, each at a time. TBR1 is predominantly expressed by layer VI neurons, CTIP2 by layer V neurons, and BRN2 by layer II/III neurons, respectively. The approach allowed us to identify neurons of either fluorescent marker and genotype and, if positive for one of the cortical layer markers, assess whether they were also correctly positioned within their respective layer.

**Fig. 2:**
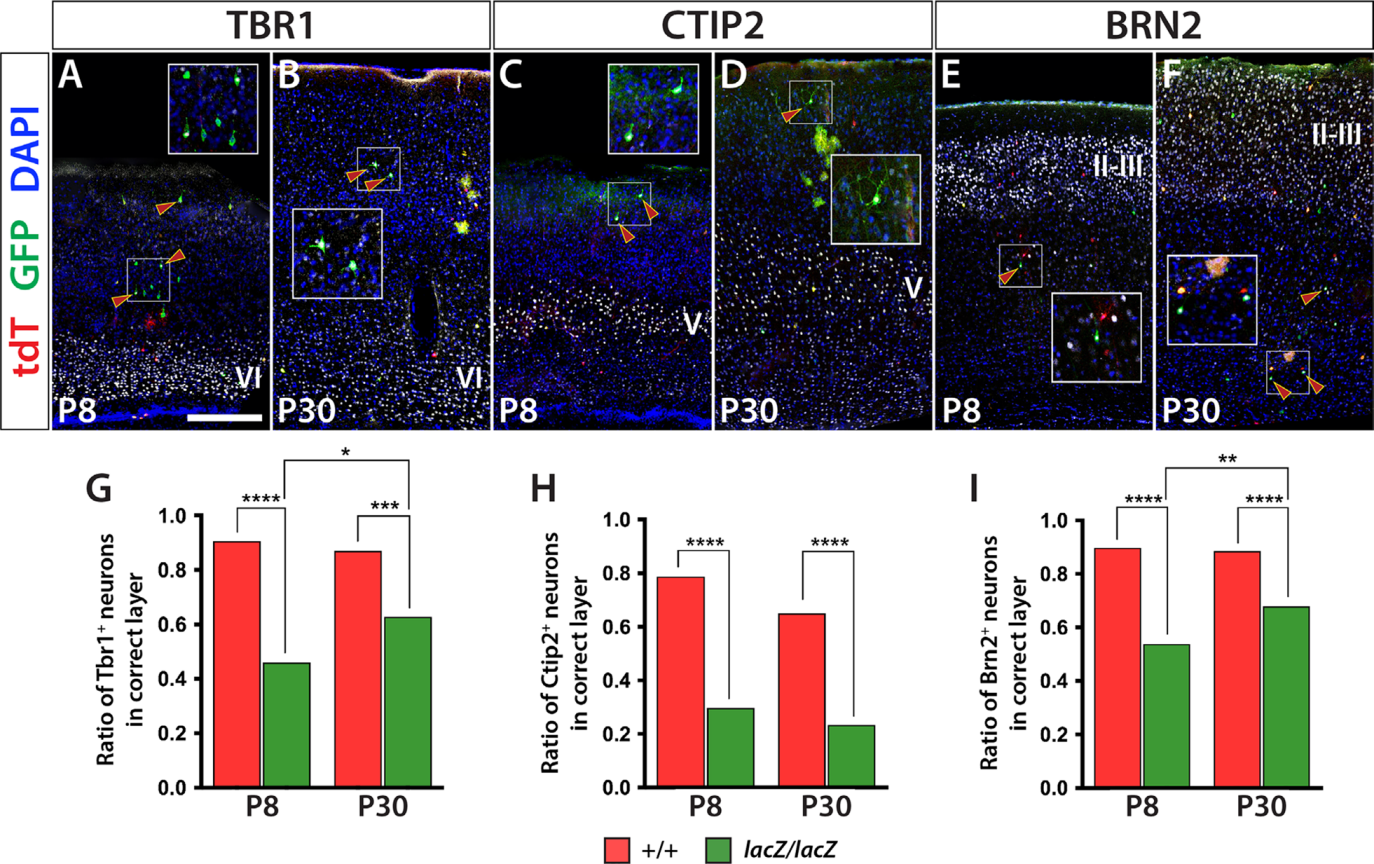
Laminar distribution of labeled neurons reveals disproportionate misplacement of *lacZ/lacZ* neurons in *Wdfy3*-MADM-5 cortex. Immunofluorescent analysis reveals laminar positioning of WT and *lacZ/lacZ* neurons in relation to layers VI (Tbr1, **A**, **B**), V (CTIP2, **C**, **D**), and II-III (BRN2, **E**, **F**). **G**–**I** Quantifications of double-labeled cells reveal that, compared to WT, a significantly smaller percentage of *lacZ/lacZ* neurons is correctly positioned in their respective layer. The proportion of correctly placed layer VI (**G**) and layers II-III (**I**) *lacZ/lacZ* neurons significantly increases between P8 and P30, likely due to loss of misplaced neurons during early postnatal development. TBR1^+^/tdT^+^ vs. TBR1^+^/GFP^+^ at P8 *p* ≤ 0.0001 and at P30 *p* ≤ 0.001. CTIP2^+^/tdT^+^ vs. CTIP2^+^/GFP^+^ at P8 *p* ≤ 0.0001 and at P30 *p* ≤ 0.0001. BRN2^+^/tdT^+^ vs. BRN2^+^/GFP^+^ at P8 *p* ≤ 0.0001 and at P30 *p* ≤ 0.0001. Scale bar is 250 μm

Analyzing a total number of 730 cells of six brains (3 male, 3 female) at P8, mutant neurons in *Wdfy3*-MADM mice were mispositioned more often than their WT counterpart. TBR1^+^ (VI) mutant cells were correctly positioned in 46% of instances compared to ~ 91% of WT cells (WT 90.66%; *lacZ/lacZ*, 46%; *p* < 0.0001) (Fig. 2A, G). Mutant CTIP2^+^ cells (V) were also significantly less often correctly positioned compared with WT cells (WT, 78.79%; *lacZ/lacZ*, 29.76%; *p* < 0.0001) (Fig. 2C, H). The same observation was made with BRN2^+^ cells (II/III) with mutant neurons being significantly less often correctly located compared with WT neurons (WT, 89.78%; *lacZ/lacZ*, 53.91%; *p* < 0.0001) (Fig. 2E, I). Examining layer-specific positioning of 735 neurons in six *Wdfy3*-MADM brains at P30 (3 male, 3 female), we found similar, but less exaggerated discrepancies between WT and *lacZ/lacZ* neurons in P8 brains. TBR1^+^ mutant cells were aligned in the correct layer in ~ 63% of instances, compared to 87% for their WT counterpart (WT, 86.81%; *lacZ/lacZ*, 62.82%; *p* = 0.0003) (Fig. 2B, G). CTIP2^+^ mutant cells showed correct layer position ~ 23% of the time compared with of ~ 65% WT cells (WT, 65.06%; *lacZ/lacZ*, 23.38%; *p* < 0.0001) (Fig. 2D, H). Lastly, BRN2^+^ mutant neurons were placed in the correct layer ~ 68% of the time, compared with ~ 89% of the time for WT cells (WT, 88.52%; *lacZ/lacZ*, 67.98%; *p* < 0.0001) (Fig. 2E, I).

Comparing the two stages (P8 and P30) with each other, the proportion of correctly aligned TBR1^+^ mutant neurons increased from ~ 46% at P8 to ~ 63% at P30 (*p* = 0.0338). Comparably, BRN2^+^ mutant neurons with correct layer position increased from ~ 54% in P8 brains, to ~ 68% in P30 brains (*p* = 0.0095), while CTIP2^+^ mutant neurons did not show significant differences between the two developmental time points (*p* = 0.3787). Also, no significant differences were present for WT neurons of any marker between the two stages and no significant deviations between the sexes were recorded. Combining the data of all three markers together, ~ 45% of mutant neurons showed correct laminar placement in P8 brains, increasing to ~ 59% in P30 brains (P8, 44.87%; P30, 58.58%; *p* = 0.0003). This relative increase likely reflects the selective elimination of mispositioned *lacZ/lacZ* neurons during the examined postnatal period.

### *Wdfy3* mutant pyramidal neurons show decreased dendritic arborization

Neurodevelopmental disorders, including autism, have been linked with changes in neuronal morphology [44, 45]. In the MADM system, sparse labeling and the fact that cells are entirely filled by the either fluorescent protein (eGFP or tdT) provided us with the option to examine cellular morphological parameters and compare them between genotypes. We focused in particular on the complexity of dendritic arbors and the density of dendritic spines or boutons. To capture labeled neurons to the greatest possible extent, 200-μm-thick sections of three male and three female agarose-embedded *Wdfy3*-MADM brains were prepared and immunostained for eGFP and tdT applying a free-floating protocol. Subsequently, sections were cleared by means of the ultrafast optical clearing method (FOCM) [42] and mounted on microscope slides for deep imaging of labeled cortical pyramidal neurons. Acquired z-stacks were traced to create Sholl profiles of 81 upper layer (II/III) and 81 deep-layer (V/VI) cortical projection neurons of either genotype.

**Fig. 3:**
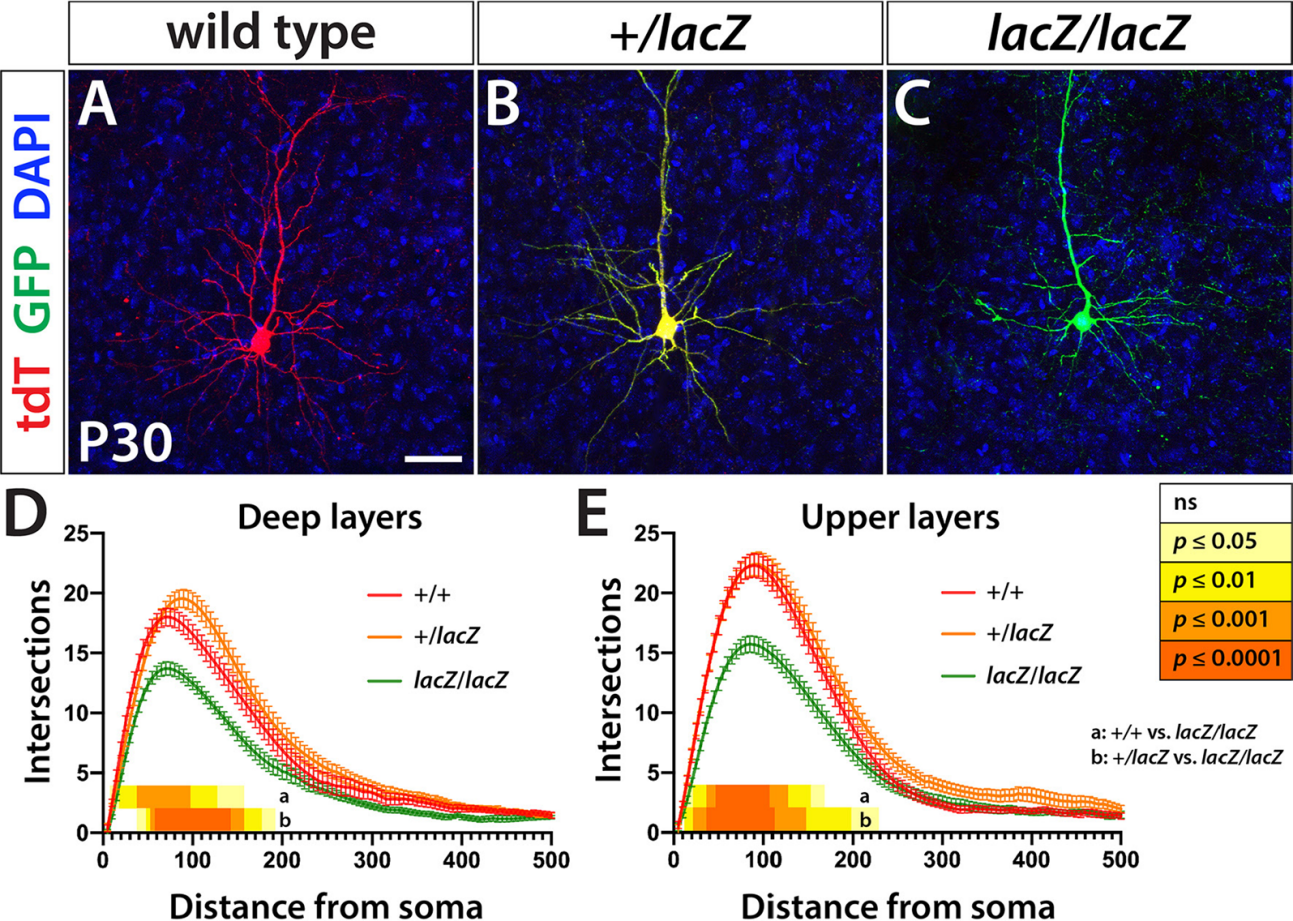
*Wdfy3* loss leads to reduced dendritic arborization. **A**–**C** TdT and GFP immunofluorescence of individual neurons shows changes in dendritic arbor complexity. **D**, **E** Statistical analyses of Sholl profiles confirm significant changes of both deep and upper layer WT (+/+) or heterozygous neurons (+/*lacZ*) and *lacZ/lacZ* neurons. Significant deviations are indicated by the heatmaps adjacent to the x-axes of the Sholl profiles and *p* values resolved on the key on the right. Scale bar is 100 μm

For either upper layer or deep-layer neurons, both WT and heterozygous cells were more complex than homozygous mutant cells, while no noteworthy differences were observed between sexes. For each genotype, the mean number of intersections peeked at ~ 100 μm from the soma and significant differences between genotypes were mostly confined to distances of 15 μm to 200 μm from the soma. The number of maximum intersections for deep-layer WT neurons at ~ 100 μm was in average 18 while *lacZ/lacZ* neurons showed a peak average of 13.7 intersections (*p* < 0.05—0.0001). Heterozygous neurons were slightly but not significantly more complex than WT with an average of 19.5 intersections at ~ 100 μm distance from the soma (+ */lacZ* vs. *lacZ/lacZ*, *p* < 0.05—0.001) (Fig. 3D). In upper layers, WT and + */lacZ* neurons showed near identical Sholl profiles with peak intersections at ~ 100 μm being on average 22.3, while *lacZ/lacZ* neurons showed a highest average of 15.7 intersections at ~ 100 μm from the soma (WT or + */lacZ* vs. *lacZ/lacZ*, *p* < 0.05—0.0001) (Fig. 3E).

### *Wdfy3* mutation increases synaptic spine density

To assess dendritic spine density and subtype distribution, 200-μm sections of four male and three female brains were immunolabeled and cleared via FOCM as described above. From acquired z-stacks of 42 labeled cells of each genotype, 10-μm (in z-depth) regions of dendrites were then extracted and dendritic spines manually counted while also tracking morphological features associated with widely described subtypes, including filopodia, long thin, thin, stubby, mushroom, and branched [46].

A decrease in *Wdfy3* gene dosage led to an overall increase in spine density. In WT neurons, spine density averaged 0.29 spines per 1 μm. In heterozygous neurons, density significantly rose to an average of 0.42 spines per μm. In *lacZ/lacZ* cells, we recorded an even higher density of 0.54 spines per μm, while no significant sex- or layer-specific differences (II/III vs. V) were observed in any genotype leading us to combine all counts. (WT, 0.2907 ± 0.1111; + */lacZ*, 0.4188 ± 0.1004; *lacZ/lacZ* 0.5409 ± 0.1609; WT vs. + */lacZ p* = 0.0286; WT vs. *lacZ/lacZ p* = 0.0011; + */lacZ* vs. *lacZ/lacZ*; *p* = 0.0287.)

**Fig. 4:**
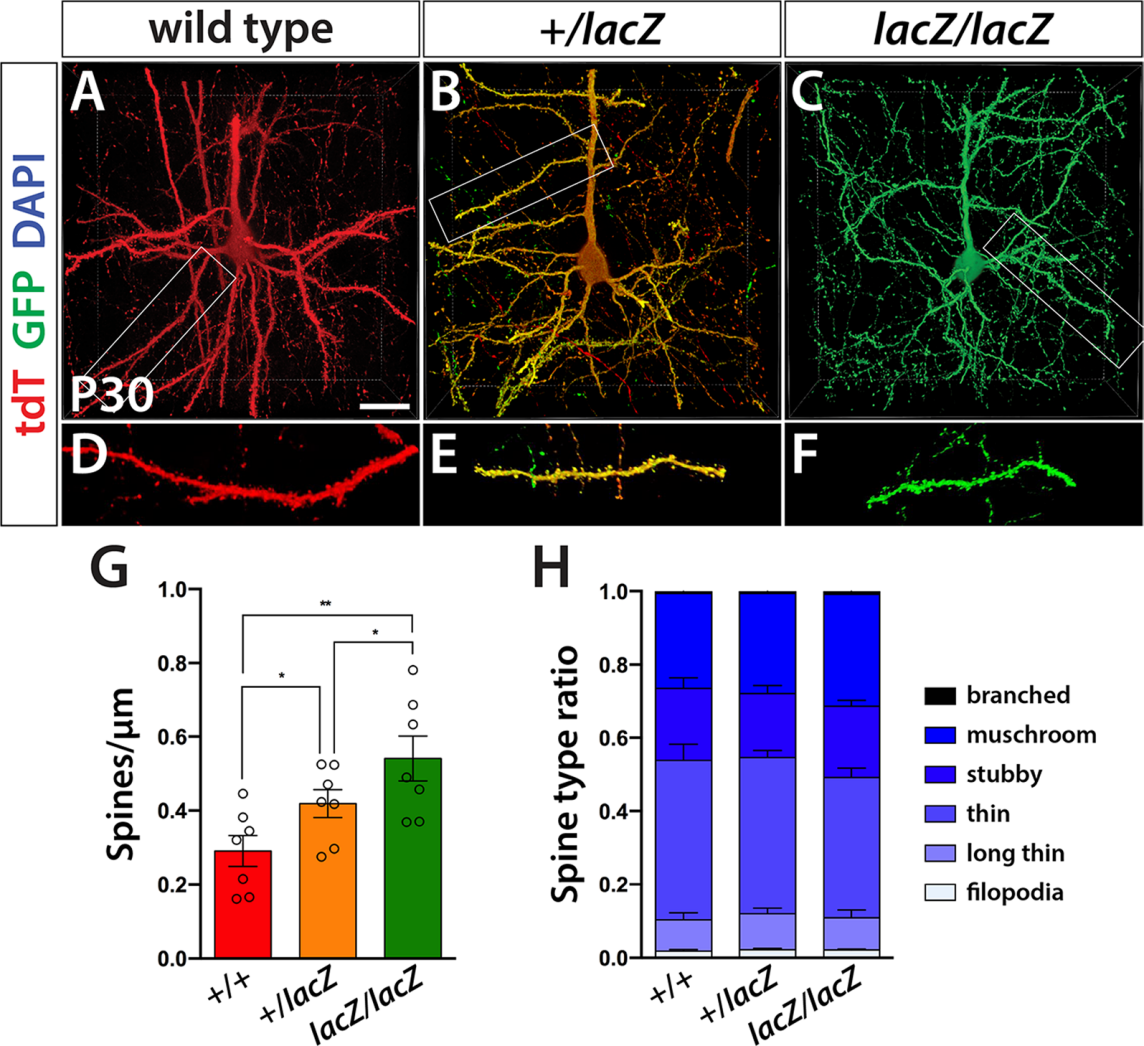
Dendritic spine density increases with progressive *Wdfy3* loss. **A**–**C** Representative 3D images of tdT and GFP immunolabeled neurons that are either wild type (**A**), heterozygous (**B**), or homozygous (**C**) mutant. **D**–**F** High-magnification images of individual dendritic spines. **G** Quantified and statistically analyzed spine density (mean ± SD) in basal and apical dendrites after the first bifurcation. Both + /*lacZ* (*p* ≤ 0.05) and *lacZ/lacZ* (*p* ≤ 0.01) neurons show significantly higher bouton density compared with WT. Homozygous mutant neurons also display higher bouton density compared with heterozygous neurons (*p* ≤ 0.05). **H** Bar diagram displaying the relative distribution of dendritic spines types found in analyzed cortical neurons. No significant differences were noted between genotypes with respect to spine subtype distribution. Scale bar is 20 μm

Dendritic spines can be distinguished by morphological criteria that associate with their state of maturation and function. A well-replicated rank order of maturation distinguishes spines that are filopodia, long thin, thin, stubby, mushroom shaped, and branched [46]. While assessing overall spine density, we traced these different subtypes and converted counts to a percentage of a whole to analyze distribution across genotypes. No significant differences between genotypes were detected from a two-way analysis of variance (ANOVA), followed by a Tukey’s multiple comparison test (Fig. 4H). Irrespective of genotype, the most prevalent spine types were long thin, thin, and mushroom, comprising ~ 90% of all counted spines. In summary, we found *Wdfy3* mutation to affect spine density in a gene dosage-responsive manner, but to have no effect on spine subtype distribution.

## Discussion

Human *WDFY3* mutation will result in neurodevelopmental disorders, such as intellectual disability, neurodevelopmental delay, and most frequently ASD associated with macrocephaly [26–30, 32]. While the megalencephalic phenotype has been faithfully replicated in both heterozygous and homozygous *Wdfy3* mutant mice and its origins traced to altered RGC divisions [24, 32], the developmental origins of the focal neuronal migration defects of homozygous mutant mice remain uncertain. Migration defects of cortical projection neurons can have either intrinsic causes or be the result of disrupted cell–environment interactions [4, 47, 48]. For instance, predominantly intrinsic mechanisms underlie lissencephaly caused by mutations in *LIS1* (*PAFAH1B1*) or *DCX* [49, 50]. LIS1 interacts with cytoplasmic dynein, a molecular motor required for microtubule-based transport [51, 52]. *Lis1* knockdown in mice will block interkinetic nuclear migration, a requirement for both RGC divisions and the motility of neuronal precursors that will derive from them [53]. Similarly, mutations in neuronal tubulin *TUBA3* (*Tuba1* in mice) will cause classic lissencephaly [33]. Migration defects rooted in the loss of extrinsic signals have been extensively studied in *reeler* mice that carry a mutation in *reelin* (*Reln*) [54, 55], a secreted glycoprotein produced by Cajal–Retzius cells occupying the marginal zone during development [56]. Reln signaling will activate an intracellular cascade of pathways that will promote migration and correct positioning of cortical neurons critical components of which are Notch signaling [57] and cadherin-mediated cell adhesion [58].

During mouse cortical neurogenesis, WDFY3 is predominantly expressed by dividing RGCs, the pia, and diffusely in the intermediate zone [24]. By using *Emx1*^*Cre*^ as a driver, this study sought to isolate cell autonomous vs. non-cell autonomous effects of WDFY3 loss on radial migration by conditionally limiting mutation to cortical projection neurons and their progenitors. Our results strongly suggest the predominance of intrinsic WDFY3 activity guiding migration and possibly survival of cortical neurons in the developing cortex. Indeed, while at P8 WT cells overwhelmingly localize within the cortical layers specific to their marker identity, *lacZ/lacZ* cells do so at significantly reduced frequency, typically at only half the rate of WT cells. At P30, we found the ratio of *lacZ/lacZ* neurons that are correctly positioned within layers VI (TBR1^+^) and II/III (BRN2^+^) to be elevated compared to P8. This finding very likely points to corrective mechanisms that are designed to eliminate misplaced and/or inadequately circuit-integrated neurons from the developing brain via programmed cell death. Indeed, TUNEL analysis of mouse parietal neocortical fields 1, 3, and 40 (largely corresponding to the area examined in the present study) revealed cell death to peak at P4, but also to continue well into the third postnatal week [43]. These findings are supported by earlier studies that recorded in rodents an average of 30% neuronal loss during the first postnatal month [59]. More recent work, however, describes a lesser, approximately 12% loss of excitatory neocortical neurons, confined to the period between P2 and P5 [60]. In light of our own data, it is conceivable that this period may be extended in case pathological dysregulations require expanded corrections. That early postnatal neuronal apoptosis is controlled by electrical activity appears also strongly supported by elegant work that associated electrical activity patterns with neuronal death rates in two distinct cortical areas (M1 and S1) [61]. Thus, the suggestion that misplaced neurons may be especially targeted for removal as their incorporation into physiologically functional networks is less likely, plausibly explains our observations of progressively fewer lacZ/lacZ neurons located outside their correct layers.

Homozygous mutant neurons display reduced dendritic arborization compared with the other genotypes. While interpretation of this finding remains speculative, possible explanations may be the loss of WDFY3-mediated functions required for cellular physiology and dendritic function. As previously reported, WDFY3 supports optimal cellular bioenergetics via mitophagy and glycophagy [62, 63]. An alternative explanation may center around a possible WDFY3 requirement for cytoskeletal organization, as widespread axonal defects in homozygous mutants suggest (unpublished observations and [64]). Interestingly, decreased dendritic complexity in *Wdfy3* mutant neurons agrees with findings in other ASD risk genes [65–69], has been observed postmortem in the hippocampi of autism cases [70], and may be a point of convergence between ASD and Rett syndrome [44].

Progressive *Wdfy3* gene loss results in concomitant increase in synaptic spine density. This finding agrees with previous reports of added dendritic spine density in layer V pyramidal neurons in the temporal lobe of ASD cases [71]. Apparently, augmented spine density in ASD is the result of reduced developmental spine pruning precipitated by hyperactivated mTOR signaling and resulting autophagy impairment [71]. Weakened autophagic function appears to be the molecular cause of reduced spine elimination in ASD cases, as well as in *Tsc1/2*- and *Atg7*-deficient cortical projection neurons, as analysis of mutant mice confirmed, drawing intriguing parallels to WDFY3 and its well-established role in regulating selective macroautophagy. While previous results did not show diminished autophagic flux in brains of *Wdfy3* mutant mice, these studies were either conducted in embryonic [24] or perinatal stages of homozygous mutants [64] and, due to perinatal lethality of homozygous *Wdfy3* mutation, only in heterozygous adults [62]. Thus, subtle changes in autophagy function caused by the synergy of homozygous *Wdfy3* loss and progressed age may have eluded detection. Of course, it is possible that greater spine density in *Wdfy3* mutant cells is caused by autophagy-independent mechanisms that currently remain elusive.

*Wdfy3*-MADM produced the predicted sparse labeling with WT red and *lacZ/lacZ* green cells being generated in the same ratio, while + */lacZ* yellow cells were twice as frequent as homozygous genotypes. The rates of labeled cells are consistent with past findings and the expectation that yellow cells being born out of G2-Z events, G1 recombination, and postmitotic recombination (G0) will amount to approximately double as many cells as either red or green cells [37, 38, 41]. The fact that yellow heterozygous cells are born at different timepoints poses difficulties in comparing phenotypic features of this genotype to the other genotypes. However, we noted that at least with respect to spine density heterozygous cells assumed an intermediate phenotype between WT and homozygous mutant cells. This finding indicated dosage sensitivity which may be relevant to human disease pathogenesis that is typically associated with haploinsufficiency. In these situations, additional, conceivably environmental, factors may be responsible for full expression of the autistic phenotype [72–75].

Whether migration defects are present in typically heterozygous *WDFY3* patients remains uncertain as of now. While MRI data for some patients are available with no evidence of focal cortical dysplasia [32], limitations in resolution cannot provide certainty that small-scale lamination defects do not occur and may influence the clinical presentation in these patients. Our work in mice found no mispositioned neurons in heterozygous mutants, but is also facing the constraint that only two hypomorphic alleles have been examined in this respect, much fewer than the number of known human alleles (49 alleles listed in denovo-db and 13 alleles reported in [32]). Potential lamination defects have not been analyzed in other available *Wdfy3* mutant mouse lines [64].

## Limitations

*Wdfy3* mutant mice have been a valuable tool in understanding prenatal aspects of ASD pathology that remain inaccessible to investigation in humans. However, like most animal models they are not a perfect model in replicating all aspects of human ASD biology, as ASD-relevant behavioral deviations in these mice remain inconclusive as of yet. It is also uncertain whether alterations in neuronal morphology described here apply to ASD patients, considering the greater complexity of the human brain and the lack of patient data.

## Conclusions

In humans, *WDFY3* is a recognized autism risk gene associated with macrocephaly. *Wdfy3* mutations in mice produce a complex phenotype of cortical maldevelopment including focal neuronal migration defects of excitatory neurons. Using MADM technology in this study, we arrive at the conclusion that these migration errors are driven by cell autonomous mechanisms that act directly on mutant neurons and their progenitors rather than impaired cell–environment interactions. In addition, we discovered alterations in mutant neuronal morphology consistent with other reports documenting decreased dendritic arbor complexity but increased spine density in autism mouse models and human cases. Our findings further underline the validity of *Wdfy3* mutant mice as ASD models and point to the significance of changes at the chemical synapse in ASD neuropathology.

## Supplementary Information

**Additional file 1. Fig S1.** Cell death analysis at P8 by CAS3 immunofluorescence.

## Data Availability

Datasets analyzed and experimental tools such as transgenic mice used in the current study are available from the corresponding author on reasonable request.

## Abbreviations

ANOVA: Analysis of variance
ASD: Autism spectrum disorders
GFP: Green fluorescence protein
VZ: Ventricular zone
FOCM: Ultrafast optical clearing method
MADM: Mosaic analysis with double markers
SVZ: Subventricular zone
DAPI: 4′,6-Diamidino-2-phenylindole
tdT: Tandem dimer Tomato
WDFY3: WD repeat and FYVE domain containing 3
WT: Wild type
