# Correction: *WDFY3* mutation alters laminar position and morphology of cortical neurons

**DOI:** 10.1186/s13229-023-00539-4

**Published:** 2023-01-31

**Authors:** Zachary A. Schaaf, Lyvin Tat, Noemi Cannizzaro, Alexios A. Panoutsopoulos, Ralph Green, Thomas Rülicke, Simon Hippenmeyer, Konstantinos S. Zarbalis

**Affiliations:** 1 Department of Pathology and Laboratory Medicine, University of California at Davis, Sacramento, CA 95817 USA; 2 Shriners Hospitals for Children Northern California, Sacramento, CA 95817 USA; 3 Department of Physiology and Membrane Biology, University of California at Davis, Sacramento, CA 95817 USA; 4 Department of Biomedical Sciences, University of Veterinary Medicine Vienna, 1210 Vienna, Austria; 5 Institute of Science and Technology Austria, Am Campus 1, 3400 Klosterneuburg, Austria; 6 UC Davis MIND Institute, Sacramento, CA 95817 USA

**Correction : Molecular Autism (2022) 13:27**. 10.1186/s13229-022-00508-3

Following publication of the original article [1], the authors reported that the 4th author (Alexios A. Panoutsopoulos) was omitted from the author group.

The author group has been updated above and the original article [1] has been corrected.
