# Supplementary material for: WDFY3 mutation alters laminar position and morphology of cortical neurons
Source: Mol Autism. 2022 Jun 22;13:27. doi: 10.1186/s13229-022-00508-3 (PMC9219247; doi:10.1186/s13229-022-00508-3)
Supplement: Supplementary file 1 — Additional file 1. Fig S1. Cell death analysis at P8 by CAS3 immunofluorescence. [file 13229_2022_508_MOESM1_ESM.docx]

**Supplementary Figure**


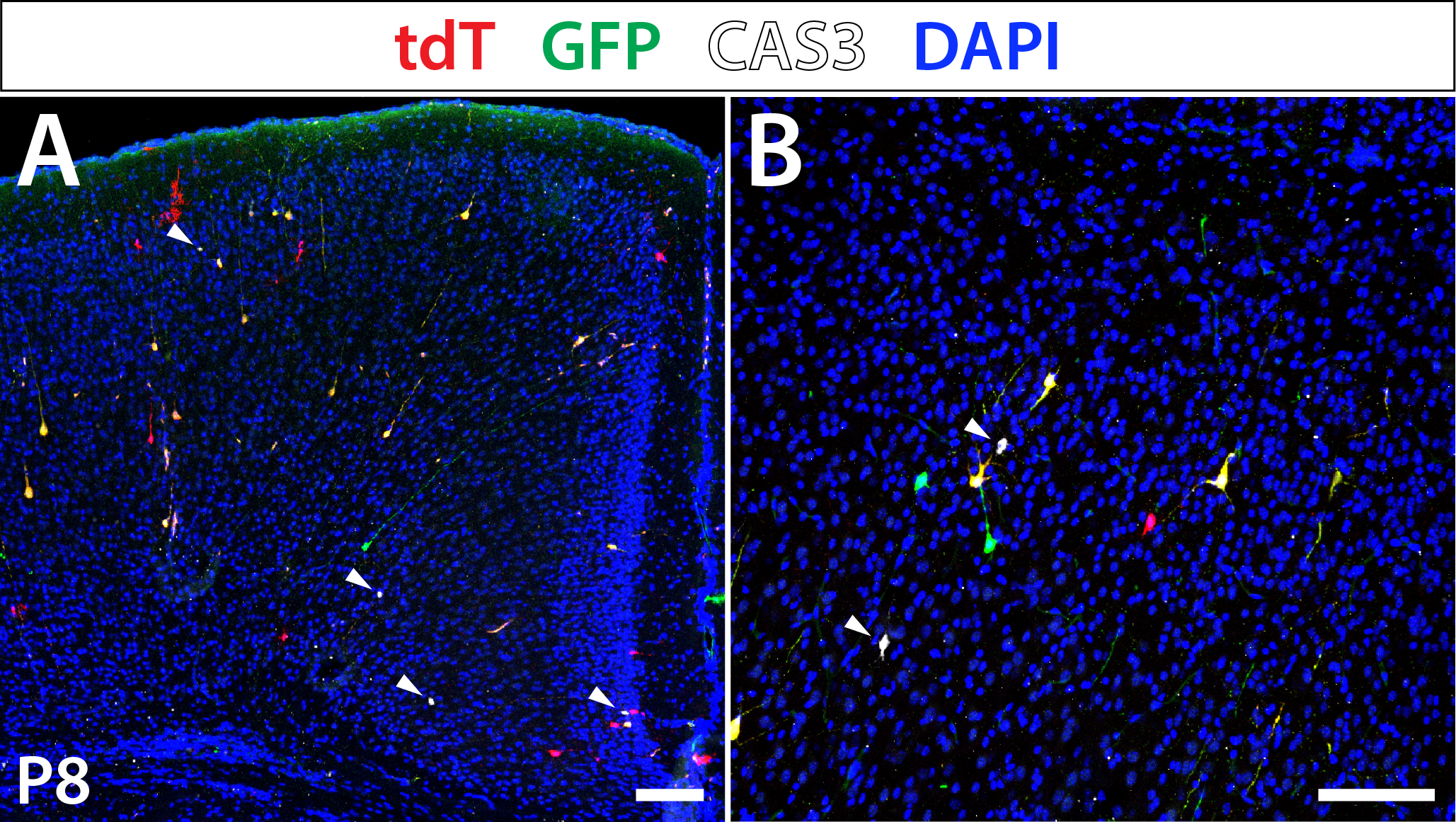


**Figure S1. Cell death analysis at P8 by CAS3 immunofluorescence**

(**A**, **B**) Representative images at 10x (**A**) and 20x (**B**) of combined tdT, GFP, and cleaved caspase 3 (CAS3) immunofluorescence in P8 cortex. No cells can be identified in which genotype-specific signals overlap with the Cas3 signal. White arrowheads point at CAS3^+^ neurons. Scale bars are 100 μm.
